# Eco-Friendly Low-Cost Design of Superhydrophobic Cu Mesh for Efficient Oil–Water Separation

**DOI:** 10.3390/molecules31111966

**Published:** 2026-06-05

**Authors:** Meizi Tian, Hong Zhao, Yanyan Liu, Ge Liu, Xiaogang Guo

**Affiliations:** 1 School of Robot Engineering, Yangtze Normal University, Chongqing 408100, China; 2 Chongqing Key Laboratory for New Chemical Materials of Shale Gas, College of Chemistry and Chemical Engineering, Yangtze Normal University, Chongqing 408100, China

**Keywords:** metal-based mesh, eco-friendly design, oil–water separation, excellent stability

## Abstract

Promising network materials with controllable porosity and tunable structures have demonstrated numerous advantages in oil–water separation applications. However, existing preparation methods generally have problems such as complex processes and adverse environmental impacts. Therefore, inspired by lotus leaves and rose petals, we have successfully designed an efficient oil–water separator based on copper meshes using in situ chemical etching, environmentally friendly fatty acid modification, and mild microwave curing treatment. Characterization results from FESEM, EDX, and XRD demonstrate that the product has high purity and a relatively uniform structure. In addition, this efficient oil–water separator has low surface energy, high hydrophobicity, and excellent oil–water separation efficiency (>98%). Moreover, after aging tests, the product has excellent structural stability and repeatable recyclability. Therefore, this research provides a convenient, cost-effective, and environmentally friendly approach for designing feasible superhydrophobic metal mesh-based devices, highlighting their wide application potential in treating industrial oily wastewater.

## 1. Introduction

In recent years, oil spill incidents and related industrial activities have become increasingly frequent. The catastrophic damage they inflict on local ecosystems goes far beyond a surface slick; the impacts cascade and amplify along a “physical–chemical–biological–economic–social” chain, generating enormous costs and evolving into a systemic crisis that spans multiple dimensions and decades [1,2,3,4]. Consequently, purifying oily wastewater is more urgent than ever, and rapid, efficient oil–water separation carries profound significance. Several conventional separation techniques, including centrifugation, sedimentation, adsorption, in situ burning, air flotation, and biological treatment [5,6,7,8,9,10,11], have been explored to treat oil-contaminated water, using various materials (e.g., polymers [12], nano-fiber membranes [13], CO_2_-responsive membranes [14]). However, the further large-scale application of these technologies is limited by factors such as the complexity of the processes, high costs, and the potential for secondary pollution.

It is noteworthy that copper mesh materials are garnering increased scholarly attention for oil–water separation owing to their cost-effectiveness and operational simplicity [15,16]. X. Gong et al. reported a superhydrophobic copper mesh via anodizing and impregnation, which exhibited a high oil separation flux (32,330 L/(m^2^·h)) and efficiency (97%) [17]. Another copper mesh coated by robust superhydrophilic/superoleophobic paint based on fluorine-containing epoxy resin was proven to have excellent mechanical properties and oil–water separation ability [18]. Y.R. Xu’s group designed ZnO nanorod arrays/polyvinylidene fluoride/octadecylamine composite membranes on copper mesh combining magnetron sputtering, hydrothermal and impregnation methods. For the oil (dichloromethane)–water mixtures tested, the membrane separation efficiency was outstanding, exhibiting favorable physical and chemical stability [19]. Furthermore, it has been proven that coating copper mesh with different layers (graphene oxide (GO)/AgBr [20], a metal Ni core, and a polar NiO/Ni(OH)_2_ shell [21], or organosilica nano/microstructures [22]) offers significant advantages and excellent separation performance in the oil–water separation process. Despite these promising advances, critical challenges still hinder the large-scale practical application of copper mesh-based oil–water separation materials, including reducing the process complexity and keeping the preparation process at a low cost. Therefore, it is extremely urgent to develop technologies that combine convenient preparation, low cost, and good separation effects. Furthermore, many existing technologies involve the introduction of fluorides. It is worth noting that excessive fluoride intake harms diverse organs and systems, and fluoride contamination has become a key contributor to environmental degradation, with fluoride pollution in any form emerging as a grave problem [23,24,25]. Therefore, designing non-fluorinated, environmentally friendly, and cost-effective technologies for oily wastewater treatment remains a significant challenge.

In order to solve the aforementioned problems, starting from the molecular design concept, we abandon traditional fluorine-containing modifying molecules and propose a convenient two-step method, namely surface micro-etching + environmentally friendly modification, to fabricate an ultra-hydrophobic copper mesh with excellent rough structure and low surface energy, and two corresponding types of oil–water separators were designed. The prepared samples exhibit excellent microstructural stability and stability in oil–water separation efficiency. Thus, the reported new concept is expected to expand the use of mesh-based materials for oil–water separation and to offer a convenient technical route for practical oil-spill remediation.

## 2. Results and Discussion

**Figure 1:**
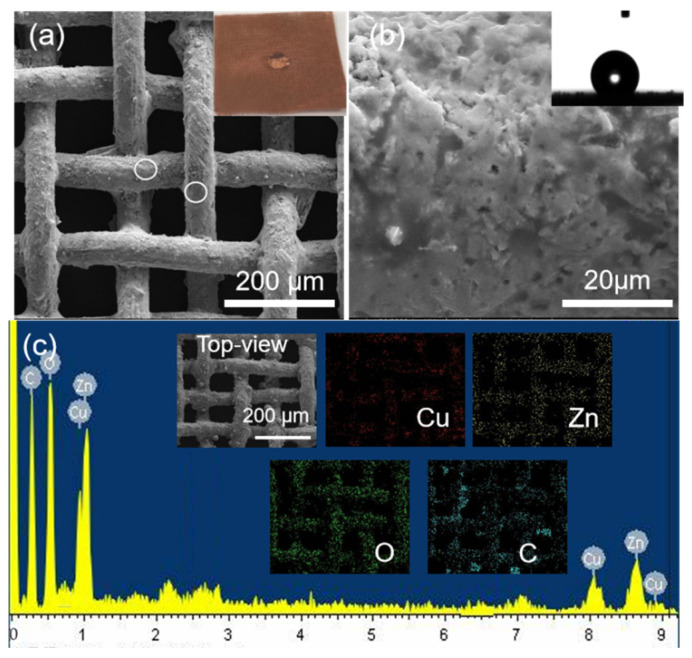
Typical FESEM images of Cu mesh sample with low (**a**) and high (**b**) resolution, followed by (**c**) the EDX spectrum and elemental distribution.

The morphologies of the Cu mesh were meticulously investigated and were characterized by field emission scanning electron microscopy (FESEM), as shown in Figure 1. Clearly, through the combination of convenient chemical etching and surface modification treatment proposed in this study, the Cu mesh surface achieved a relatively uniform structure (Figure 1a), and the droplet could rest on the copper mesh surface in a nearly perfect spherical shape, as shown in the inset image in Figure 1a. In the enlarged view, the etching treatment increased the surface roughness of the copper mesh, constructing the micro/nano hierarchical structure required for superhydrophobic surfaces. According to the Wenzel and Cassie–Baxter wetting models, the increased surface roughness amplifies the hydrophobicity of the low-surface-energy modified coating, enabling air to be trapped in the rough grooves to form a composite solid–liquid–air interface. This finally leads to a static water contact angle (CA) as high as 155 ± 1° (>150°, Figure 1b), confirming the excellent superhydrophobicity of the as-prepared sample. Furthermore, energy dispersive X-ray (EDX) spectrum and element mapping images were used to analyze the elemental distribution of the modified Cu mesh in Figure 1c. Clearly, the top view images showed Cu, Zn, C, and O as the main elements, which are uniformly distributed across the surface.

**Figure 2:**
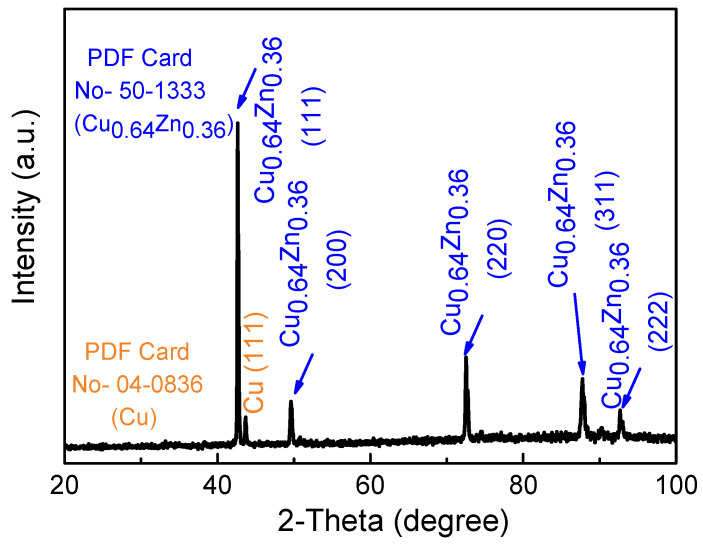
XRD analysis of as-designed Cu mesh.

In addition, the crystal structure of the sample was analyzed using X-ray diffractometer (XRD) technology. As displayed in Figure 2, all main XRD diffraction peaks were indexed as belonging to Cu_0.64_Zn_0.36_ (PDF No. 50-1333) and Cu (PDF No. 04-0836) for the fresh Cu mesh sample, which was consistent with the EDX analysis of the sample, further demonstrating that the sample has high purity with no other impurities, while also confirming the efficiency and feasibility of the used technique.

**Figure 3:**
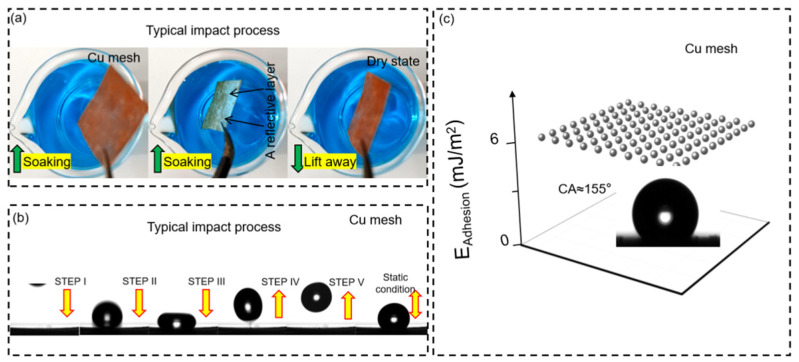
Typical immersion process (**a**) and impact process (**b**) of the Cu mesh, and the *E_Adhesion_* (**c**) of the Cu mesh.

The wettability of the sample was thoroughly investigated, as shown in Figure 3. First, the typical immersion process of the Cu mesh is displayed in Figure 3a. The soaked part of the Cu mesh sample appeared as a reflective silver mirror surface due to the presence of an adsorbed air cushion [26]. The surface of the Cu mesh after the immersion process was still totally dry, which further confirmed its outstanding hydrophobic properties. Figure 3b illustrates the process of a water droplet bouncing on the surface. This complete bouncing cycle involves five distinct stages: (i) falling, as the droplet descends towards the surface, (ii) contact, when it makes initial contact with the material, (iii) compression, experienced from the impact and interaction with the surface, (iv) detachment, when the droplet separates from the surface due to the material’s elasticity or other forces, and (v) rebounding, where the droplet bounces off the surface, possibly gaining considerable height during this sequence. Water droplets detached and rebounded from the surface of the Cu mesh quickly owing to the low energy loss during the rebounding process [27,28], which is also consistent with the high CA analysis. In addition, the adhesion energy (*E_Adhesion_*) was as low as 6 mJ/m^2^ in almost every region of the sample (Figure 3c), indicating the ultra-low adhesion of the Cu mesh to water droplets.

**Figure 4:**
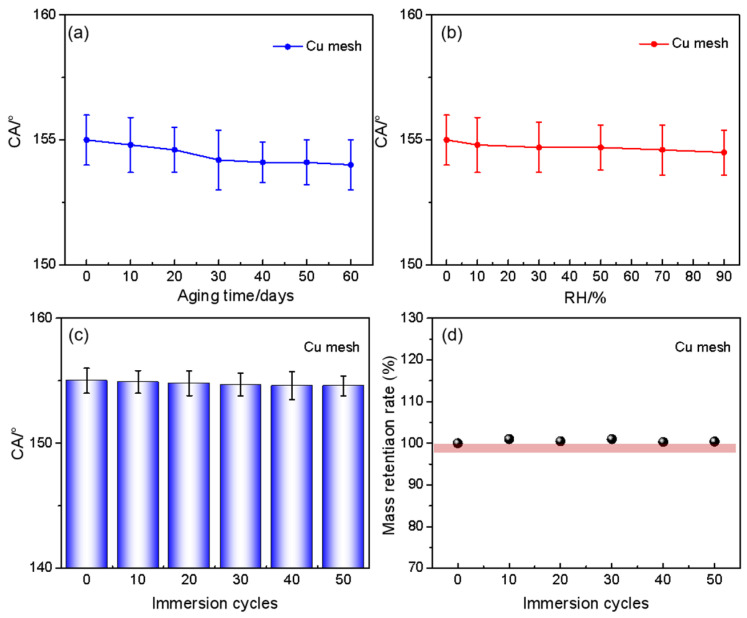
CA as functions of (**a**) aging time, (**b**) relative humidity (RH), and (**c**) immersion cycles, and (**d**) the mass retention rate (%) of sample after different immersion cycles.

Furthermore, after 30 days of aging tests, the CA kept almost stable, as shown in Figure 4a, and the effect of relative humidity on wettability was almost negligible (Figure 4b). In summary, Figure 4c depicts how immersion cycles relate to CA using five steps: contact, partial immersion, total immersion, partial lifting, and total lifting, which together form one complete cycle. The Cu mesh consistently maintained a CA of approximately 155°, demonstrating excellent superhydrophobic stability. Figure 4d depicts the mass retention rate of the sample after different immersion cycles. Clearly, even after dozens of immersion cycles, the mass of the Cu mesh sample scarcely increased, further underscoring its superiority in wetting resistance and practical applications.

**Figure 5:**
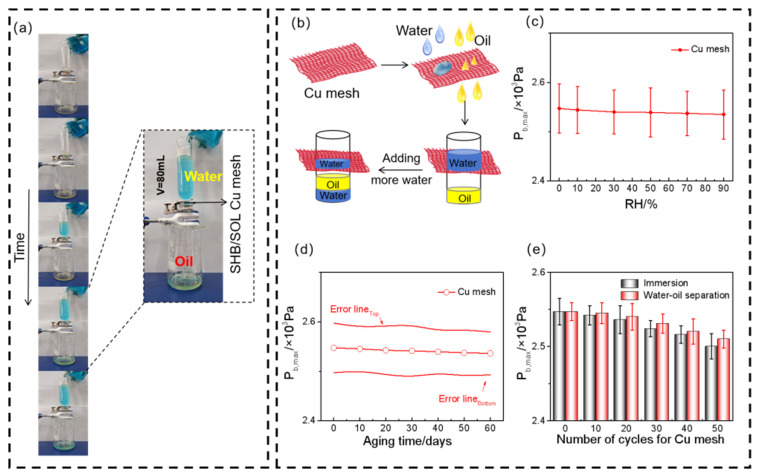
(**a**) Pressure-bearing experimental analysis for the Cu mesh, followed by the corresponding process illustration diagram (**b**), and the maximum bearing pressure (P_b, max_) as functions of (**c**) relative humidity (RH), (**d**) aging time, and (**e**) immersion and oil/water separation cycles.

To explore the practical applications of oil/water separation for the Cu mesh, a pressure-bearing experiment was conducted using gasoline and a dyed aqueous solution. Figure 5a illustrates the separation process, where a mixture of oil–water at different volumes of V = 80 mL was poured into a tube. The oil drained through the Cu mesh into the beaker, demonstrating the superhydrophobic ability of the mesh by the pressure from the mixture. As shown in Figure 5b, as the droplet of water and oil fell on the Cu mesh, it drained the oil as the water droplet rolled off the surface of the mesh. However, as more water was added to the glass jar, some water drained into the oil when the superhydrophobic resistance reached its limit at maximum bearing pressure. Figure 5c,d show the analysis of the maximum bearing pressure (P_bmax_) of the Cu mesh with its corresponding percentage in relative humidity, and to the respective aging time of 60 days in an air exposure experiment. It is evident that aging time and humidity variations have only a minor effect on the sample’s bearing pressure capacity, with a (P_bmax_) of ~2.55 kPa. In addition, although immersion and oil/water separation cycles exerted some influence on the load-bearing performance, the capacity retention rate remained above 90% (Figure 5e), demonstrating the sample’s excellent prospects for practical application.

**Figure 6:**
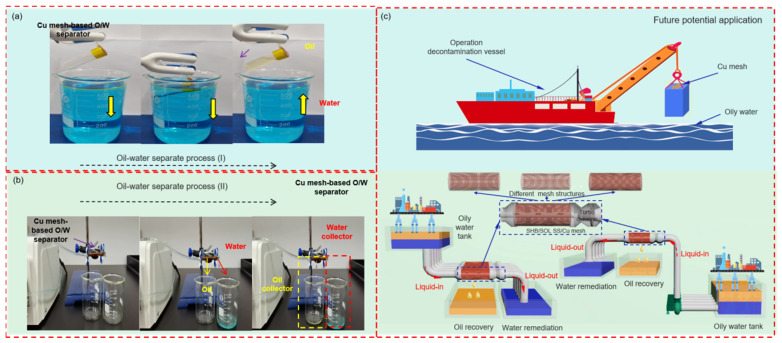
Two typical oil/water separation processes, with Cu mesh-based O/W separator moving (**a**) and (**b**) oily water flowing, followed by the corresponding future potential application (**c**).

To quantify the translational potential of the Cu mesh-based O/W separator, we engineered two complementary high-throughput O/W separation skids (Figure 6). The first setup integrates an inclined oil-collection vial on a motorized stage clamped to a standard retort stand; slow immersion and retrieval of the vial into the O/W emulsion allow gravity-driven, plug-flow separation (Figure 6a). The second skid couples the mesh to a precision peristaltic loop that continuously delivers separated oil and water into distinct reservoirs (Figure 6b). Owing to the mesh’s pronounced superhydrophobicity, the aqueous phase is rapidly expelled and channeled into the water collector, providing instantaneous validation of separation efficiency. Translating these bench metrics to open-water scenarios, Figure 6c depicts a mobile separation tank equipped with a reciprocating Cu mesh-based separator. This unit can be retrofitted onto response vessels to reclaim petroleum products from marine slicks. The same modular platform is readily upscaled for rapid remediation of oil-laden urban wastewater streams.

**Figure 7:**
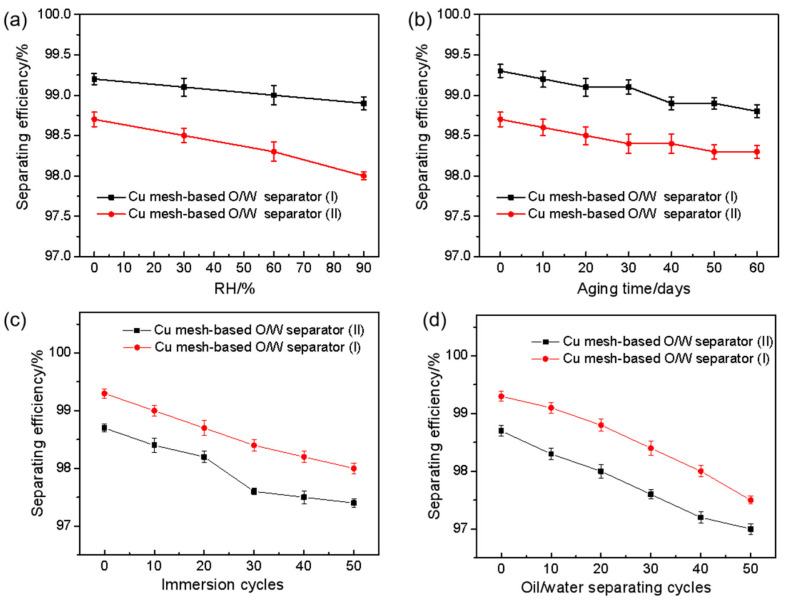
The O/W separation efficiency as functions of (**a**) RH (%), (**b**) aging time, (**c**) immersion cycles, and (**d**) oil/water separation cycles.

The influence of aging duration, ambient humidity and immersion–drain cycles on separation performance was further investigated. Figure 7a,b show the relationship between the O/W separation efficiency and the relative humidity (RH) and aging time in days, respectively. Various RH environments were used for analyzing the superhydrophobocity of the Cu mesh-based O/W separator. The separators achieved only slight decreases in separation efficiency from dry environments (RH = 0%) to moist environments (RH = 90%), and also across an aging time of 60 days. Even after prolonged aging under high humidity conditions, the meshes retained near-native O/W separation efficiency. Moreover, Figure 7c,d explain the relationship between the separation efficiency of immersion cycles and oil/water separation cycles for the Cu mesh-based O/W separator. As observed, the average O/W separation efficiency of the Cu mesh-based O/W separator remained above >97% even after 50 continuous cycles, demonstrating robust performance for practical applications. The specific cyclic experiment processes (take O/W separation process (I) as example) are recorded in detail, including the first cycle (Video S1, Supporting Information), any intermediate cycle (take the 25th as an example, Video S2), and the last cycle (Video S3). In addition, the process pictures also verify the excellent oil–water separation effect of the product (Table S1, Supporting Information). It is worth mentioning that after 50 cycles of oil–water separation, the microstructure of the copper mesh surface hardly changed (Figure 1a and Figure S1a in Supporting Information) and the crystal structure still retained similar XRD diffraction peaks (Figure 2 and Figure S1b), which further verify the structural stability of the as-obtained copper mesh. Building upon the superhydrophobic and oleophilic oil–water separation process designed using copper meshes as reported in the relevant literature [28,29,30,31], this study not only offers advantages such as operational simplicity and short processing time, but also demonstrates outstanding oil–water separation efficiency (Table 1), further validating the broad application potential of this approach.

## 3. Experimental Section

### 3.1. Reagents and Materials

Ferric trichloride (FeCl_3_) and stearic acid were purchased from Shanghai Aladdin Bio-Chem Technology Co., Ltd. (Shanghai, China). Other reagents (e.g., ethanol and HCl) were purchased from Sinopharm Chemical Reagent Co., Ltd. (Shanghai, China). The copper meshes were commercially available. Unless stated otherwise, all chemicals were used as received without additional purification, and deionized (DI, 18 MΩ·cm) water was used in this study.

### 3.2. Fabrication of Efficient O/W Separator

To prepare the efficient O/W separator, copper meshes (purity ≥ 99.9%) were precision-cut into 2 cm × 2 cm and 5 cm × 5 cm samples and manually abraded with 2000 grit SiC paper. To eliminate surface oxides, the copper meshes with different sizes were sequentially ultrasonicated in 0.1 M HCl for 10 min under 25 °C, and alternately washed with deionized water and ethanol. Then, surface roughening was performed by immersion in an etching solution composed of FeCl_3_·6H_2_O (0.12 g mL^−1^) and polyethylene glycol (2 wt % relative to FeCl_3_) at 25 °C for 20 min in a water bath pot. After etching, all samples were rinsed with deionized water and ethanol alternately, and dried at 100 °C for 10 min in a vacuum drying oven. To enhance the hydrophobicity of the sample, all treated meshes were moved into a 4.5 wt % stearic acid solution in anhydrous ethanol + isopropanol (v:v = 50:1) at 50 °C for 1 h under a sealed atmosphere for surface energy reduction, followed by vacuum heat treatment at 100 °C for 10 min and then cooled to ambient temperature. Finally, products were obtained and stored in a desiccator until further characterization.

### 3.3. Characterization

Surface morphologies and crystal structures of the O/W separator were analyzed by a FESEM (FEI Quant 250F, FEI, Hillsboro, OR, USA) with EDX spectroscopy, and an XRD (Shimadzu ZD-3AX, Shimadzu, Kyoto, Japan) equipped with monochromatized Cu Kα1 radiation (λ = 1.54178 Å), respectively. The wetting of the O/W separator surface was investigated by using a contact angle measuring instrument (SDC350, Shengding Co., Ltd., Shenzhen, China), and RH aging tests were conducted in a calibrated salt-spray chamber (LRHS-108-RFHY, LP, Shanghai, China) maintained at room temperature.

### 3.4. Oil/Water Separation

The O/W separation was conducted using efficient O/W separators (copper mesh-based devices), and two kinds of separation processes were realized by (I) the O/W separator moving, and (II) the liquid moving through a roll-like channel with the O/W separator. The separation efficiency (S_E_) was determined by Equation (1):

(1) $$S_{E}=\frac{V_{A}}{V_{B}}\times 100\%,$$

where V_A_ and V_B_ are the volume of oil before and after O/W separation using two kinds of O/W separators.

## 4. Conclusions

In brief, we have developed a low-cost fluorine-free molecular modification technique that exhibits exceptional efficacy and universal applicability using Cu mesh samples under suitable conditions to obtain the novel functional O/W separator possessing great tunable morphological, structural and anti-wetting properties with promising applications. In addition, the target Cu mesh-based O/W separator showed outstanding structural stability and superhydrophobicity, with a high CA of >155° after a 60-day exposure experiment, and the product possessed great hydrophobic stability after 50 successive immersion and bouncing cycles. The separation efficiency of the obtained separator was >97% and remained almost stable even after 50 separation cycles in a high relative humidity environment. This work introduces a new feasible path for the green molecular design of oil–water separation functional materials, and enriches the application practice of green molecular science in the field of environmental functional materials.

## Figures and Tables

**Table 1:** Comparison of O/W separation performance of copper meshes with different coated materials in the literature.

Materials	Substrate	Maximum Separation Efficiency (%)	Characteristics of the Preparation Process	Ref.
ZIF-8	Copper mesh	>97.2	Involves multiple steps and is relatively cumbersome to perform	[28]
TCMS	Copper mesh	>99.8	Process takes slightly longer	[29]
Cu_2_O/Volatile organics	Copper mesh	>96	Process takes a long time, and is relatively cumbersome to perform	[30]
Polyimine/MOF composite	Copper mesh	>99.2	Process takes a little longer	[31]
Stearic acid	Copper mesh	>99%	Time-efficient and easy to operate	This work

## Data Availability

All data sets generated and materials used during the current study are available from the corresponding author on reasonable request.

## Supplementary Materials

The following supporting information can be downloaded at: https://www.mdpi.com/article/10.3390/molecules31111966/s1, Figure S1: The microstructure (a) and XRD crystal structure (b) of the copper mesh surface after 50 O/W separation cycles; Table S1: Process snaps of multiple (1–50) oil–water separation cycles of the copper mesh; Video S1: The 1st oil–water separation cycle process; Video S2: Any single cycle process in the oil–water separation cycle experiment, taking 25 cycles as an example; Video S3: The 50th oil–water separation cycle process.
