# Supplementary material for: Eco-Friendly Low-Cost Design of Superhydrophobic Cu Mesh for Efficient Oil–Water Separation
Source: Molecules. 2026 Jun 5;31(11):1966. doi: 10.3390/molecules31111966 (PMC13258656; doi:10.3390/molecules31111966)
Supplement: Supplementary file 1 [file molecules-31-01966-s001.zip › Supporting Information.pdf]

molecules

Supporting information

for

Eco-Friendly Low-Cost Design of Superhydrophobic Cu Mesh for Efficient Oil–Water Separation

Meizi Tian [1], Hong Zhao [2], Yanyan Liu [2], Ge Liu [1] and Xiaogang Guo [2,*]

[1] School of Robot Engineering, Yangtze Normal University, Chongqing 408100, China
[2] Chongqing Key Laboratory for New Chemical Materials of Shale Gas, College of Chemistry and Chemical Engineering, Yangtze Normal University, Chongqing 408100, China
* Correspondence: guoxiaogang0528@126.com

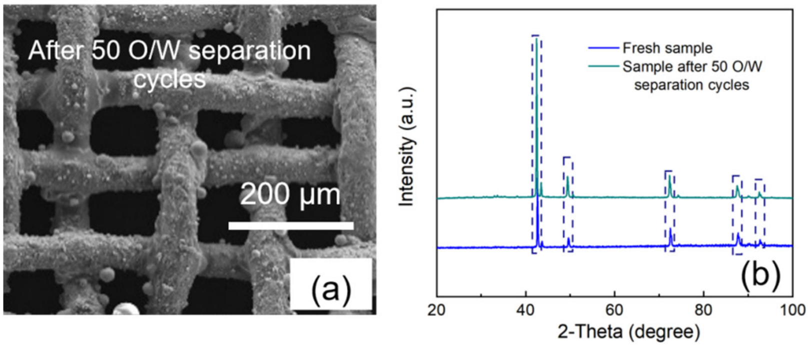

Figure S1. The microstructure (a) and XRD crystal structure (b) of the copper mesh surface after 50 O/W separation cycles.

Video S1. The 1st oil-water separation cycle process.

Video S2. Any single cycle process in the oil-water separation cycle experiment, taking 25 cycles as an example.

Video S3. The 50th oil-water separation cycle process.

 https://doi.org/10.3390/molecules31111966

Table S1. Process snaps of multiple (1-50) oil-water separation cycles of the copper mesh.

Number of cycles	Initial oil-water mixture	Separation begin	During the separation process	End of separation	Oil collection
1st	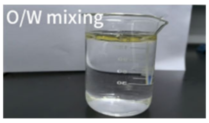	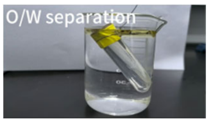	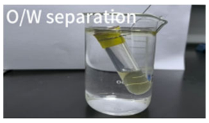	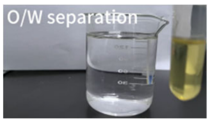	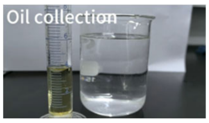
25th	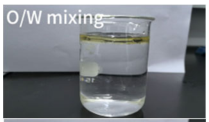	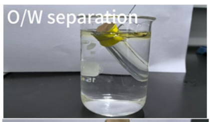	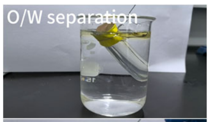	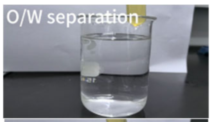	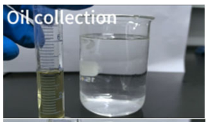
50th	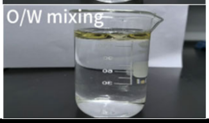	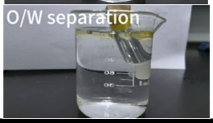	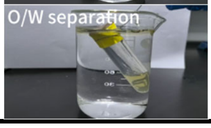	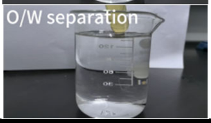	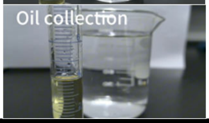

https://doi.org/10.3390/molecules31111966
